# The Prokinetic Face of Ghrelin

**DOI:** 10.1155/2010/493614

**Published:** 2010-02-10

**Authors:** Hanaa S. Sallam, Jiande D. Z. Chen

**Affiliations:** Department of Internal Medicine, Division of Gastroenterolog, University of Texas Medical Branch, Galveston, TX 77550, USA

## Abstract

This review evaluated published data regarding the effects of ghrelin on GI motility using the PubMed database for English articles from 1999 to September 2009. Our strategy was to combine all available information from previous literature, in order to provide a complete structured review on the prokinetic properties of exogenous ghrelin and its potential use for treatment of various GI dysmotility ailments. We classified the literature into two major groups, depending on whether studies were done in health or in disease. We sub-classified the studies into stomach, small intestinal and colon studies, and broke them down further into studies done in vitro, in vivo (animals) and in humans. Further more, the reviewed studies were presented in a chronological order to guide the readers across the scientific advances in the field. The review shows evidences that ghrelin and its (receptor) agonists possess a strong prokinetic potential to serve in the treatment of diabetic, neurogenic or idiopathic gastroparesis and possibly, chemotherapy-associated dyspepsia, postoperative, septic or post-burn ileus, opiate-induced bowel dysfunction and chronic idiopathic constipation. Further research is necessary to close the gap in knowledge about the effect of ghrelin on the human intestines in health and disease.

## 1. Introduction

Ghrelin is a 28-amino acid motilin-related peptide hormone mainly secreted by the X/A-like enteroendocrine cells of the oxyntic (parietal) mucosa of the gastric fundus. It is the endogenous ligand for the growth-hormone secretagogue receptor (GHS-R) 1a, recently discovered by two independent research groups [1, 2]. The discovery of ghrelin bore great importance in the scientific media for the following reasons: (1) ghrelin is the only endogenous (natural) ligand for the GHS-R, ever discovered; (2) ghrelin shares similar structure with motilin, the so-long called “orphan peptide,” based on its unique structure; (3) ghrelin is the first known case of a peptide hormone modified by a fatty acid, the *n*-octanoic acid; a step necessary for ghrelin to exert its effects [1]; (4) ghrelin is a hunger hormone that stimulates appetite, food intake and promotes weight gain, that is, ghrelin antagonist would serve as a potential treatment for obesity. In addition, ghrelin's poly-faceted properties include prokinetic and possible anti-inflammatory abilities. Although there has been several excellent reviews published recently on the prokinetic effects on ghrelin, some were brief [3, 4], or combined with motilin [5, 6], and some were focused on the effect of ghrelin on the interdigestive motility [7], gastrointestinal [GI] disorders [8, 9], or a certain subset of disease [10]. In the current review, we offer a complete detailed review solely on the prokinetic effects of exogenous ghrelin on the stomach and intestines in health and in disease.

## 2. Methods

This review evaluated published data regarding the prokinetic properties of exogenous ghrelin using the PubMed database for English articles from 1999 to September 2009. Our strategy was to combine and distil all currently available studies and reviews in order to provide an overview on exogenous ghrelin and its prokinetic abilities along the gut in health and disease. The search was performed by combining the terms “ghrelin” with “prokinetic” or with “gastrointestinal motility.” Clinical trials and review articles were specifically identified, and their reference citation lists were searched for additional publications not identified in the database searches. We classified the literature into two major groups, depending on whether studies were done in health or in disease. We sub-classified the studies into stomach, small intestinal and colon, and broke them down further into in vitro, in vivo (animals) and in humans. The reviewed studies were presented in a chronological order to guide the readers across the scientific advances in the field. We have combined all the available information from previous literature, in order to provide a complete review on the prokinetic properties of exogenous ghrelin and its potential applications for the treatment of various gastrointestinal (GI) dysmotility ailments.

## 3. Effects of Ghrelin on Gastric Motility in Health

### 3.1. In Vitro Gastric Tissues in Health

in vitro studies demonstrated the prokinetic potential of ghrelin or its (receptor) agonists in enhancing gastric muscle contractility via activation of the growth hormone secretagogue receptor (GHS-R) and direct neural stimulation of the enteric nervous system (ENSs); an effect involving the cholinergic and tachykininergic pathways. Earliest studies were done in 2003 by Dass et al. on rodent gastric fundic circular muscle strips. Ghrelin concentration dependently increased the amplitude of cholinergic off-contractions at concentrations from 0.1 to 10 *μ*M. Ghrelin did not affect the neurogenic electrical field stimulation (EFSs) induced contractions [11]. They confirmed their results in 2005 [12]. Only a year later, were they able to prove that ghrelin increased the EFS-induced contractions using a concentration of 1 *μ*M [13]. Fukuda et al. confirmed this effect on the antrum and body longitudinal muscle strips using the same concentration of 1 *μ*M [14]. Depoortere et al. showed that ghrelin and ghrelin receptor synthetic peptide agonist, growth hormone receptor peptide 6 (GHRP-6), enhanced off-contractions on both the fundus and the antrum [15]. They went further to report such effects in the presence of N_*ω*_-Nitro-_L_-arginine-methyl-ester-hydrochloride (L-NAME), to verify the involvement of nitric oxide (NO) in the EFS-induced relaxation on-response. Ghrelin or GHRP-6 also enhanced L-NAME-induced contractions; an effect that was reproducible in mice [15, 16]. The same research group confirmed the involvement of GHS-R in ghrelin's excitatory effect on gastric muscle tissue either by direct identification of the GHS-R 1a transcripts in the muscle strips, or by testing the effect of treatment with the synthetic nopeptide GHS-R agonist (capromorelin) [15, 16]. In the same year, Levin et al. [17], reported that ghrelin's excitatory effect on the gastric fundus was blocked by the pretreatment with atropine, suggesting the involvement of the cholinergic pathway. Such conclusion was confirmed a year later by Bassil et al. who showed the involvement of the tachykininergic pathway, as well [13]. Recently, ghrelin's excitatory effects have also been reported in the upper gut of birds [18, 19].

### 3.2. In Vivo—Animal Stomach in Health

In vivo studies showed the role of ghrelin in the regulation of the migrating motor complex (MMC) in the fasting state and the involvement of the activation of GHS-R and neuropeptide Y (NPY) and possibly, vagal cholinergic neurons. In 2003, Fujino et al. reported that IV ghrelin induced MMC in the antrum of vagotomized fed rats via activation of the GHS-R and NPY neurons [20]. In fact, ghrelin appears to be the endogenous signal for the MMC in rodents [21]. In 2008, Taniguchi et al. showed that ghrelin infusion increased the motility index (MI) of antral phase III-like contractions, dose dependently, in conscious freely moving rats [7]. A year later, they confirmed the same finding of increase MI in conscious freely moving mice [22]. Zheng et al. reported that ghrelin-induced phase III-like contractions in the antrum of freely moving mice are mediated via the vagal cholinergic pathway [23]. In dogs, however, ghrelin was reported to have no effect on MMCs, suggesting a possible species-related variation [24]. Such variation may be related to the fact that rodents do not express the motilin receptor, whereas in dog, expressing ghrelin and motilin receptors, it is possible that the endogenous signal for the MMC to be modulated by motilin, with no response to ghrelin. 

 In vivo studies revealed opposite effects of ghrelin on the proximal and distal gastric tone in anaesthetized rats. Kobashi et al. reported that ICV ghrelin, or direct injection of ghrelin into the dorsal vagal complex (DVC), relaxed the proximal stomach, while ICV ghrelin at a higher dose contracted the distal stomach [25]. These results suggested the presence of GHS-R in the DVC, as well as the involvement of vagal preganglionic neurons in the action of ghrelin on gastric tone. 

 In vivo studies debated the effect of ghrelin on gastric myoelectrical activity (GMA). We have previously reported no effects of ghrelin on GMA in healthy dogs [26] and have confirmed it in rats (Sallam—unpublished). However, Tümer et al. reported an enhancement of GMA following ghrelin treatment using the same dose we used in rats [27]. We tend to disagree that the reported enhancement was substantial considering that it was within 5%–10%; moreover, the rats had an almost 90% of normal slow waves before the ghrelin treatment. 

 A number of in vivo studies preceded the in vitro studies and demonstrated prokinetic effects of ghrelin on gastric motility exerted via a vagally mediated mechanism. Masuda et al. were the first to report that IV ghrelin enhanced gastric contractions dose-dependently in anesthetized rats (from 0.8, 4, and 20 *μ*g/kg) and the effect was blocked by the pretreatment with atropine or vagotomy [28]. Later, Trudel et al. however, could not reproduce the results in anaesthetized rats; instead they performed the study in conscious rats and reported that IV ghrelin dose-dependently (5 and 20 *μ*g/kg) accelerated gastric emptying [29]. A similar effect was reported with subcutaneous ghrelin (100 nmol/Kg) in conscious mice [30]. Fukuda et al. reported that IV ghrelin (20 *μ*g/kg) accelerated gastric emptying of both nutrient and non-nutrient meals in conscious rats. Pretreatment with capsaicin blocked the effect of ghrelin on only the nutrient meal [14]. Using ghrelin infusion of 500 pmol/Kg/min and a similar gastric emptying assessment method, Levin et al. could not reproduce Fukuda's results regarding ghrelin's acceleration of the nutrient meal [17]. However, a number of other studies confirmed the ability of intravenous (IV) or intraperitoneal (IP) ghrelin or ghrelin (receptor) agonists in the acceleration of gastric emptying in rats [15, 27, 31, 32] and mice [16, 32, 33], using a variety of methods for the assessment of gastric emptying. Such results were equally reproducible in ghrelin receptor knockout mice, suggesting a minor role of ghrelin in gastric emptying regulation [34]. In dogs, ghrelin was reported to show no effect on gastric emptying in one study, but excitatory effects on gastric contractions in another [24, 26].

### 3.3. In Human—Stomach of Healthy Subjects

Studies on the prokinetic effects of exogenous ghrelin on the stomach of healthy human volunteers are summarized in Table 1. Researchers have been able to reproduce some of the in vitro and in vivo results in healthy humans. The effects of ghrelin on the fasting, not fed, human stomach was similar to in vivo studies. In fasted volunteers, ghrelin induced phase III-like contractions [35]; an effect that was confirmed two years later by Bisschops [36]. However, both research groups, in addition to Cremonini et al. [37], reported that ghrelin increased the tone of the proximal stomach in the fasting state in healthy volunteers. Even postprandially, ghrelin increased the tone of the proximal stomach [38]. Such result is contradictory to an in vivo study in which ghrelin decreased proximal stomach tone in rats [25]. However, it is noteworthy that this particular in vivo study was performed in anesthetized, not conscious animals. 

 In 2006, researchers debated the prokinetic effect of ghrelin on gastric emptying [37, 39]. It is possible that the use of different techniques for assessment of gastric emptying might have been responsible for this discrepancy. 

 Ghrelin was reported to inhibit gastric accommodation in healthy volunteers [38]. This raises the question of whether ghrelin would induce side effects, similar to erythromycin, the macrolide antibiotic and motilin receptor agonist that inhibits postprandial accommodation and induces postprandial symptoms. However, ghrelin has been shown not to affect meal related symptoms in healthy obese or lean subjects [37, 38]. This is conceivable, as healthy volunteers would not be expected to have pronounced postprandial symptoms.

## 4. Effects of Ghrelin on Gastric Motility in Disease

### 4.1. In Vitro Gastric Tissues in Disease

Two recent studies by Qiu et al. reported that ghrelin or GHRP-6 increased the amplitude of carbachol-induced contractions of the gastric fundus of various diabetic rodent models [40, 41]. These results were reproducible in vivo (see below).

### 4.2. In Vivo Gastric Motility in Animal Model of Diseases

Studies on the prokinetic effects of exogenous ghrelin on diseased stomach in vivo are summarized in Table 2. A number of studies have reported that ghrelin or GHRP-6 accelerated or normalized gastric emptying in a variety of diseased animal models; these include: diabetic, postoperative, or morphine, or septic, or burn induced ileus, and cisplatin induced dyspepsia models. (1)* Diabetic model*: Qiu et al. published 3 papers in 2008 confirming their in vitro findings in two diabetic rodent models [40, 41, 47]. They also found that the excitatory effects of ghrelin or GHRP-6 on gastric emptying in diabetic gastroparesis were mediated via the cholinergic pathway. (2)* Postoperative ileus and/or morphine-treated model*: In 2002, Trudel et al. showed that IV ghrelin (20 *μ*g/kg) normalized gastric emptying in a postoperative ileus rat model [29]. A year later, they confirmed the same finding in dogs [42]. In 2005, the same group reported that RC-1139, a ghrelin receptor agonist, accelerated gastric emptying, dose-dependently, in postoperative ileus or in healthy morphine-treated rodent model. However, when postoperative ileus animals were also given a dose of morphine, a higher dose of RC-1139 was needed to accelerate gastric emptying [31]. In 2007, Venkova et al. tested another ghrelin receptor agonist, TZP-101 and showed its ability to accelerate gastric emptying in a postoperative ileus rodent model whether or not it was aggravated by morphine [45]. (3)* Septic model*: in 2004, De Winter et al. reported that IP ghrelin or GHRP-6 accelerated gastric emptying in a lipopolysaccharide (LPS)-induced septic ileus rat model [33]. In 2009, Chen et al. confirmed that ghrelin accelerated gastric emptying in a LPS septic ileus mouse model, but at a much lower dose [46]. (4)* Burn model*: in 2007, we have reported the ability of ghrelin to normalize gastric emptying in a 60% total body surface area (TSBA) rat model; an effect mediated by the cholinergic pathway [44]. (5)* Cisplatin-induced dyspepsia model*: in 2006, Liu et al [43]. reported that IP ghrelin improved gastric emptying in a mouse model of dyspepsia.

### 4.3. In Human—Patients with Gastric Motility Disorders

Studies on the prokinetic effects of exogenous ghrelin in dyspeptic and/or gastroparetic patients are summarized in Table 3. From 2005 till present, most of the researchers seem to agree that ghrelin can accelerate gastric emptying in dyspeptic and/or gastroparetic patients. Unlike animal studies, the effects of ghrelin on gastric emptying in these patients were irrespective to vagal contribution [49, 50], giving hope to neuropathy gastroparetic patients. In 2009, Ejskjaer et al. reported that TZP-101, the ghrelin receptor agonist, also accelerated gastric emptying in diabetic patients with gastroparesis [51].

Though the effect of ghrelin on patients' symptoms remained debatable [36, 48, 49], TZP-101 was reported to show no effect on the postprandial symptoms of diabetic gastroparesis patients and its safety profile has been determined in a phase I trial [51, 52]. 

 Despite the optimistic results of acute ghrelin administration on gastric motility in patients with gastroparesis, many researchers showed their concern regarding the side effects of the chronic use of ghrelin. Being the ligand of growth hormone secretagogue receptor, ghrelin has been shown to induce growth hormone secretion [53] and insulin resistance [54]; such effects would cause serious unfavorable side effects in particular subsets of patients. Modification of ghrelin (receptor) agonists might be necessary to avoid such problems. Long term studies will show whether the ghrelin receptor would be desensitized by chronic activation, similar to the motilin receptor. Treatment alteration between ghrelin and motilin receptor agonists has been suggested [3]. The new ghrelin receptor agonist, TZP-101, is promising as it did not induce growth hormone secretion following either peripheral or central administration [53]. TZP-101 has also been claimed to have a lower tendency to provoke ghrelin receptor desensitization [5, 52, 55]. 

## 5. Effects of Ghrelin on Intestinal Motility in Health

### 5.1. In Vitro—Intestinal Tissues in Health

In the small intestine, studies reported a prokinetic effect of ghrelin on jejunal contractility in rodents, involving direct activation of the GHS-R on the myenteric neurons and the cholinergic pathway. In 2004, Fukuda et al. reported that ghrelin enhanced EFS-induced contractions in longitudinal jejunal muscles [14] and Edholm et al. reported that ghrelin enhanced acetylcholine-induced contractions in circular jejunal muscles; an effect mediated via the cholinergic pathway [56]. In 2008, Bisschops proved that not only ghrelin, but GHRP-6, dose-dependently activated the myenteric neurons by eliciting a Ca^2+^ transient; this depended on direct activation of the GHS-R [36]. 

 In the colonic tissues, studies have shown that the prokinetic effects of ghrelin were species-specific. While it induced colonic contractions in fish or birds [19, 57], ghrelin had no effect on the colon of rodents or humans [11, 12].

### 5.2. In Vivo—Intestinal Motility in Healthy Animals

In vivo studies showed that ghrelin induced intestinal MMCs in fed rats involving the activation of the cholinergic pathway, and the NO, NPY or 5-hydroxytryptamine 4 (5-HT_4_) receptors. In 2003, Fujino et al. reported that ghrelin induced MMCs in fed and/or vagotomized rats; this effect was blocked by immunoneutralization of the NPY receptor [20]. In 2004, Edholm reported that ghrelin dose-dependently shortened the intestinal MMC cycles. Pretreatment with atropine blocked the ghrelin effect [56]. In 2007, Wang et al. reproduced the exact same results in rats and showed that the NO and 5-HT pathways might be involved in the action of ghrelin on intestinal interdigestive motility [58]. One year later, Taniguchi et al. pinpointed the involvement of the 5-HT_4_ receptor in the ghrelin's action on intestinal MMCs [7]. Recently, using a manometric method for simultaneous assessment of gastro-duodenal motility, Tanaka et al. showed that ghrelin induced MMCs in fed conscious freely moving mice [22]. In dogs, however, ghrelin was shown to have no effect on intestinal MMCs [24]. 

 Using a variety of techniques for the assessment of intestinal transit, researchers proved that ghrelin or its receptor agonist accelerated intestinal transit in rodents. In 2002, Trudel et al. showed that IV ghrelin 20 *μ*g/kg accelerated intestinal transit in rats [29]. This prokinetic effect of ghrelin on intestinal transit has been confirmed by several researchers using IV or IP ghrelin using the same, or different dose in rats [14, 15] or mice [32, 46]. Ghrelin receptor agonists GHRP-6 or EX-1314 has also been shown to have similar prokinetic results. EX-1314 did not accelerate the intestinal transit in ghrelin receptor knockout mice, confirming the necessity of GHS-R activation for the prokinetic action of ghrelin, in accordance to what has been shown in the in vitro studies [15, 32, 36]. 

 As for the colon, studies showed that to induce colon propulsion in conscious, not anesthetized, rats, central administration of ghrelin was needed. Although, ghrelin may exert an indirect effect on the colon merely by triggering upper GI MMCs, such possibility was not evidenced in the literature. The lack of direct effect of ghrelin on the colon may possibly be due to the lack of ghrelin immunoreactive cells and/or ghrelin receptors in the colon [1, 59, 60]. In 2002, Trudel et al. reported no effect of IV ghrelin on the colon of conscious rats [29]. In 2005, Tebbe et al. showed that ghrelin, injected in the paraventricular nucleus (PVN), stimulated colonic motility, dose dependently, in freely moving conscious rats. This effect involved central activation of the PVN via the corticotrophin releasing factor 1 (CRF_1_) and the NPY_1_ receptors [61, 62]. In 2006, Shimizu et al. reported that intrathecal ghrelin increased the colonic propulsive function in anesthetized rats. Shimizu et al. reported similar effects with intrathecal, or IV, or IV infusion of CP464709, a synthetic ghrelin receptor agonist which exerted defecation in conscious rats that was dependent on intact pelvic nerves [63]. In 2009, Charoenthongtrakul et al. reported that oral EX-1314 increased fecal output in conscious mice [32]. Shafton et al. tested a centrally acting ghrelin receptor agonist, GSK894281 that was administrated orally in conscious rats. They reported a dose-dependent increase in the fecal output both acutely and after 8 days of treatment [64].

### 5.3. In Human—Intestines of Healthy Subjects

Tack et al. reported premature intestinal phase III contractions following IV ghrelin in healthy volunteers. These contractions were of gastric origin [35]. Although ghrelin, like motilin, triggered intestinal MMCs in humans, but unlike motilin, plasma ghrelin has not been reported to fluctuate with phase III, suggesting that motilin remains the main hormone dominating interdigestive motility in man. 

No studies were found on the effects of ghrelin on colon motility in healthy subjects.

## 6. Effects of Ghrelin on Intestinal Motility in Disease

### 6.1. In Vitro—Intestinal Tissues in Disease

No studies were found on the effects of ghrelin on small intestinal tissues obtained from diseased animal models. 

 However, ghrelin effects on colitis rodent model were reported. Recently, De Smet et al. showed that ghrelin decreased the colonic inhibitory responses in healthy mice and aggravated colitis in a dextran sodium sulfate (DDS)-induced colitis mice model [65]. This is contradictory to several studies in which ghrelin exerted an anti-inflammatory effect in animal models of colitis [66, 67]. Further investigation is needed to explore the role of ghrelin in colitis-induced dysmotility.

### 6.2. In Vivo—Intestinal Motility in Animal Model of Diseases

Studies on the prokinetic effects of exogenous ghrelin on diseases intestines in vivo are summarized in Table 4. Similar to its effects on gastric emptying, ghrelin or its receptor agonists have been reported to accelerate or normalize intestinal transit in a variety of diseased animal models; these include diabetic, postoperative, or morphine, or septic, or burn induced ileus, and opiate-induced bowel disorder models. (1)* Diabetic model*: Zheng et al. showed that ghrelin or GHRP-6 increased intestinal transit; this effect was mediated via the cholinergic pathway [47]. (2)* Postoperative ileus and/or morphine-treated model*: Venkova et al. tested another ghrelin receptor agonist, TZP-101, that was also shown to accelerate gastric emptying in postoperative ileus rodent model whether or not it was aggravated by morphine [45]. (3)* Septic model*: controversial results have been reported with IP ghrelin in LPS-induced septic ileus rodent models. While De Winter et al. showed that ghrelin or GHRP-6 (100 *μ*g/kg) had no effect in septic rats [33], Chen et al. showed that ghrelin (20 *μ*g/kg) normalized the intestinal transit in septic mice [46]. (4)* Burn model*: in 2007, we have reported that ghrelin normalized the intestinal transit in a 60% TSBA rat model; this effect was mediated via the cholinergic pathway [44]. (5)* Opiate-induced bowel disorder model*: recently, Charoenthongtrakul et al. reported that EX-1314 normalized opiate-induced delayed intestinal transit in mice [32]. 

 As for the colon, studies showed that IV administration of ghrelin agonists (TZP-101, ipamorelin, or GHRP-6) accelerated the colon transit in a postoperative ileus rat model [68, 69], while IP ghrelin had no effect on colon motility in a scald burn rat model [44].

### 6.3. In Human—Patients with Intestinal Motility Disorders

No studies were found regarding the effects of ghrelin in patients with intestinal motility disorders.

## 7. Summary and Conclusion

In conscious animals, exogenous ghrelin was reported to (1) induce gastric and intestinal MMCs in fed rodents, but not in the canine; (2) exert controversial effects on gastric myoelectrical activity in rodents; (3) induce antral contractions in dogs; (4) accelerate gastric emptying in healthy, diabetic, postoperative, or morphine, or septic, or burn-induced ileus, and cisplatin-induced dyspepsia animal models; (5) accelerate intestinal transit in healthy, diabetic, postoperative, or morphine, or septic, or burn induced ileus, and opiate-induced bowel disorder rodent models; (6) accelerate colonic transit in healthy rodents, when centrally administrated. 

 Clinically, exogenous ghrelin was reported to (1) induce gastric and intestinal MMCs in fasted healthy subjects; (2) increase fundic tone in both fasted and fed healthy subjects; (3) exert controversial effects on gastric emptying and have no effect on postprandial symptoms in healthy subjects; (4) accelerate gastric emptying in dyspeptic and/or gastroparetic patients and have debatable effects on postprandial symptoms in these patients. Luckily, the prokinetic effects of ghrelin in gastroparesis and/or dyspepsia patients were independent of vagal involvement. 

 The prokinetic effects of ghrelin on GI motility involve the exclusive activation of the GHS-R 1a receptor, not the motilin receptor, the enteric nervous system (specifically the myenteric plexus), excitatory neurons involving 5-HT4 and NO, capsaicin-sensitive afferent neurons, tachykininergic motor neurons, as well as intact vagal cholinergic neurons. 

 Oral ghrelin use is limited due to its instant inhibition by the gastric acidic milieu; however, other routes for its administration are possible. The emergence of IV ghrelin agonists (e.g., synthetic peptide GHRP-6; synthetic non-peptide capromorelin or ipamorelin) or ghrelin receptor agonists (e.g., GSK894281, EX-1314, EX-1315, RC-1139, TZP-101) are paving the way for possible uses in patient treatment. Oral TZP-102 soon followed and has been tested in healthy volunteers [70]. Other oral agonists include TZP-102, EX-1314 and RC-1141. 

 In conclusion, the prokinetic face of ghrelin enables it to serve as a strong tool in the clinical practice for the treatment of various GI dysmotility ailments. The prokinetic properties of ghrelin or its (receptor) agonists have the potential to serve in the treatment of diabetic, neurogenic or idiopathic gastroparesis and possibly, chemotherapy-associated dyspepsia, postoperative, septic or post-burn ileus, opiate-induced bowel dysfunction and chronic idiopathic constipation. Further research is necessary to close the gap in knowledge about the effect of ghrelin on the human intestines in health and disease.

## Figures and Tables

**Table 1:** Prokinetic effects of exogenous ghrelin on gastric motility in healthy human volunteers (D-Lys). GHRP-6: GHRP receptor antagonist; GE: gastric emptying; GHRP-6: ghrelin secretagogue receptor 6 (non-synthetic ghrelin receptor agonist); GHS-R: growth hormone secretagogue receptor; IV: intravenous; MI; motility index; MMC: migrating motor complex; SPECT: single photon emission computed tomography; VAS: visual analogue scale.

Author	Subjects	Study design	Ghrelin type	Effective dose	Methods	Results
Levin F., [39]	Healthy volunteers (5M, 3F)	Randomized, double-blind, placebo-controlled, crossover	Ghrelin	10 pmol/Kg/min for 180 min after meal	Assessment of solid GE by scintigraphy	Ghrelin accelerated the rate of GE

Tack J., [35]	Healthy fasting volunteers (4F, 5M)	Cross-sectional	Ghrelin (Clinalfa, Switzerland)	40 *μ*g IV infusion over 30 min given 20 min after the end of phase III of MMCs	Assessment of antroduodenal motility and gastric tone by manometry and barostat	Ghrelin induced premature phase III contractions and increased the tone of the proximal stomach

Cremonini F., [37]	Healthy volunteers: obese (5M, 20F) and	Randomized, parallel-group, one dose, double-blind, placebo-controlled	Ghrelin (Clinalfa, Switzerland)	0.33 *μ*g/Kg IV given 10 minutes after IV injection of ^99m^Tc-pertechnetate	Assessment of gastric volume and emptying by SPECT	Ghrelin marginally decreased fasting gastric volumes, but not GE or symptoms
	normal weight (13F)				Assessment of symptoms by VAS	

Bisschops R., [36]	Healthy volunteers (*n* = 9)	No information	(i) Ghrelin	40 *μ*g IV infusion over 30 min given 20 min after the end of phase III of MMCs	Assessment of antroduodenal pressures by manometry and gastric tone by barostat	(i) Ghrelin induced phase III contractions of gastric origin; those were of increased amplitude and duration.
			(ii) GHRP-6(Clinalfa, Switzerland)			(ii) Ghrelin increased proximal gastric tone

Ang D., [38]	Healthy volunteers (4M, 6F)	Randomized, placebo-controlled, double-blind, cross over	Ghrelin	40 *μ*g IV infusion over 30 min given10 min before meal	Assessment of gastric accommodation by barostat	(i) Ghrelin inhibited gastric accommodation and decreased postprandial gastric volumes
						(ii) Ghrelin had no effects on postprandial symptoms

**Table 2:** Prokinetic effects of exogenous ghrelin on diseased stomach in vivo. GE: gastric emptying; GHRP-6: ghrelin secretagogue receptor 6 (non-synthetic ghrelin receptor agonist); h: hour; iNOS: inducible nitric oxide synthase; IP: intraperitoneal; IV: intravenous; L-NAME: N_*ω*_-Nitro-_L_-arginine-methyl-ester-hydrochloride; NO: nitric oxide; Postop: postoperative; SD: Sprague Dawley.

Author	Species	Ghrelin type	Effective dose	Methods	Results	Mechanism of action
Trudel L., [29]	Conscious postop ileus model (SD male rats)	Human ghrelin-28 (IGBMC, France)	20 *μ*g/kg IV given immediately after meal	Assessment of GE by gastric retention of a phenol red-marked meal	Ghrelin reversed the postop delayed GE	

Trudel L., [42]	Conscious postop ileus model (female mongrel dogs)	Ghrelin (IGBMC, France)	(i) 100 *μ*g/kg IV on day 2	Assessment of GE by acetaminophen method	Ghrelin reversed the postop delayed GE	
			(ii) 4 *μ*g/kg IV on day 3			
			(iii) 20 *μ*g/kg IV on day 4given after meal			

De Winter B., [33]	Conscious LPS septic ileus model (Swiss OFI mice)	(i) Rat ghrelin (Tocris, UK)	(i) Ghrelin: 100 *μ*g/kg	Assessment of GE by the gastric retention of an Evans blue-marked meal	Ghrelin and GHRP-6 accelerated GE in LPS septic ileus mice	
		(ii) GHRP-6 (Bachem, UK)	(ii) GHRP-6: 20 and100 *μ*g/kg given IP 1h prior to meal			

Poitras P., [31]	Conscious postop ileus ± morphine-treated rat model (male SD rats)	Ghrelin receptor agonist RC-1139 (Rejuvenon Corp., USA)	2.5–10 mg /Kg IV given immediately after meal	Assessment of GE by the retention of ^99m^Tc-labelled meal	(i) RC-1139 accelerated GE dose-dependently in postop ileus rats	
					(ii) RC-1139 at 10 mg/Kg accelerated GE in postop ileus + morphine-treated rats	

Liu Y., [43]	Conscious Cisplatin-treated adult male C57/6J black mice	Rat ghrelin (Bachem Ltd, UK)	1 mg/Kg, IP b.i.d	Assessment of gastric emptying by the wet weight of gastric content	Ghrelin improved GE	

Sallam H., [44]	Conscious scald-burned model (SD male rats)	Ghrelin (Tocris, USA)	2 nmol/rat given IP 20 min before meal	Assessment of GE by gastric retention of a phenol red-marked meal	Ghrelin accelerated GE; an effect blocked by pretreatment with atropine	Ghrelin's effects on gastric motility involve the cholinergic pathway

Venkova K., [45]	Conscious postop ileus ± morphine-treated rat model (male SD rats)	Ghrelin receptor agonist TZP-101 (Tranzyme Pharma Canada)	0.1–1 mg /Kg (1ml) IV given 1-2 min before meal	Assessment of GE by the retention of ^99m^Tc-labelled meal	TZP-101 accelerated GE dose-dependently in postop ileus rats ± morphine	

Qui et al. [40]	Diabetic mouse model (IP-alloxan-treated C57 mice)	Rat ghrelin GHRP-6(Tocris, UK)	50–200 *μ*g/Kg given IP prior to meal	Assessment of GE by gastric retention of a phenol red-marked meal	Ghrelin and GHRP-6 at all doses accelerated GE; an effect blocked by atropine or L-NAME.	The effects of Ghrelin and GHRP-6 on GE in diabetic gastroparesis is mediated via the cholinergic pathways

Qui et al. [41]	Diabetic guinea pig model (IP-STZ-treated)	Ghrelin GHRP-6	20, 50 and 100 *μ*g/Kg given IP prior to meal	Assessment of GE by gastric retention of a phenol red-marked meal	Ghrelin and GHRP-6 at all doses accelerated GE; an effect blocked by atropine.	The effects of Ghrelin and GHRP-6 on GE in diabetic gastroparesis are mediated via the cholinergic pathways

Chen Y., [46]	LPS endotoxemia mouse model (male ICR mice)	Rat ghrelin (Global Peptide Services, UDA)	20 *μ*g/Kg IP given 15 min before meal	(i) Assessment of GE by gastric retention of a phenol red-marked meal	(i) Ghrelin had no effect on GE	Ghrelin's effect on LPS-delayed GE are mediated via the down regulation of NO
				(ii) Assessment of plasma NO production by fluorometry	(ii) Ghrelin normalized endotoxemia-induced delayed GE	
				(iii) Assessment of iNos expression by immunohistochemistry	(iii) Ghrelin 20 *μ*g/Kg reduced plasma NO and iNOS expression in the submucosa and musculosa of the stomach	

Zheng Q., [47]	Diabetic mouse model (IP-alloxan-treated C57 mice)	GHRP-6 (Tocris, UK)	200 *μ*g/kg given IP prior to meal	Assessment of GE by gastric retention of a phenol red-marked meal	GHRP-6 accelerated diabetic-induced delayed GE; an effect blocked by pretreatment with atropine	GHRP-6 effects on gastric motility involve the cholinergic pathway

**Table 3:** Prokinetic effects of exogenous ghrelin in dyspeptic and/or gastroparetic patients. IDDM: insulin-dependent diabetes mellitus; GE: gastric emptying; GHRP-6: ghrelin secretagogue receptor 6 (non-synthetic ghrelin receptor agonist); GHS-R: growth hormone secretagogue receptor; h: hour; IV: intravenous; VAS: visual analogue scale.

Author	Subjects	Study design	Ghrelin type	Effective dose	Methods	Results
Tack J., [48]	Six dyspeptic patients (5F, 1M)	Cross-sectional	Ghrelin (Clinalfa, Switzerland)	40 *μ*g IV over given 30 min at the start of the meal	(i) Assessment of GE rates for solids and liquids by the ^14^C octanoic acid and ^13^C glycin breath tests	Ghrelin accelerated GE for both liquids and solids, as well as meal-related symptom scores
					(ii) Assessment of the intensity of 6 meal-related symptoms	

Murray C., [49]	Ten IDDM gastroparetic patients (5M, 5F)	Randomized, double blinded, cross-over	Synthetic human ghrelin (Bachem, UK)	5 pmol/Kg/min IV over 2 h	(i) Assessment of GE rate by real time ultrasonography	Ghrelin accelerated GE rate, but had no effect on patients' symptoms, despite impaired cardiovagal tone
					(ii) Assessment of the symptoms by VAS	

Binn M., [50]	Six gastroparetic patients (all F; 1 with truncal vagotomy)	Cross-sectional	Synthetic human ghrelin (Merck Biosciences, Switzerland)	20 *μ*g/ml IV over 1 minute	Assessment of GE by C^13^-octanoic acid breath test	Ghrelin accelerated gastroparetic-induced delayed GE, even despite vagotomy

Bisschops R., [36]	Dyspeptic patients with delayed GE (*n* = 6)	No information	(i) Ghrelin	40 *μ*g IV infusion over 30 min given 20 min at the start of the meal	(i) Assessment of GE by ^14^C octanoic acid and ^13^C glycin breath test	(i) Ghrelin accelerated GE of liquids significantly and of solids marginally
			(ii) GHRP-6(Clinalfa, Switzerland)		(ii) Assessment of meal-related symptoms	(ii) Ghrelin decreased the cumulative meal-related symptom scores

Ejskjaer N., [51]	Diabetic patients with gastroparesis (5M, 5F)	Randomized, double-blind, placebo-controlled, single dose, cross-over	TZP-101 (Tranzyme Pharma)	80, 160, 320 and 600 *μ*g/Kg IV over 30 min after meal	Assessment of GE by scintigraphy	TZP-101 accelerated GE of both liquid and solid components of the meal; no significant effect on symptoms

**Table 4:** Prokinetic effects of exogenous ghrelin on intestinal motility in vivo in disease. CT: colon transit; GHRP-6: ghrelin secretagogue receptor 6 (non-synthetic ghrelin receptor agonist); h: hour; ICV: intracerebrovascular; iNOS: inducible nitric oxide synthase; IP: intraperitoneal; IT: intestinal transit; IV: intravenous; LPS: lipopolysaccharide; NO: nitric oxide; NPY: neuropeptide Y; Postop: postoperative; SC: subcutaneous; SD: Sprague Dawley.

Author	Species	Ghrelin type	Effective dose	Methods	Results	Mechanism of action
De Winter B., [33]	Conscious healthy and LPS septic ileus model (Swiss OFI mice)	(i) Rat ghrelin (Tocris, UK)	(i) Ghrelin: 100 *μ*g/kg	Assessment of IT by the transit of an Evans blue-marked meal	Ghrelin and GHRP-6, at either dose] had no prokinetic effect on IT in healthy or diseased mice.	
		(ii) GHRP-6 (Bachem, UK)	(ii) GHRP-6: 20 and100 *μ*g/kg IP 1h prior to meal			

Sallam H., [44]	Conscious scald-burned model (SD male rats)	Ghrelin (Tocris, USA)	2 nmol/rat given IP 20 min before meal	(ii) Assessment of IT and CT by the transit of a phenol red-marked meal	Ghrelin accelerated IT but had no effect on CT	Ghrelin's effects on intestinal motility are mediated via the cholinergic pathway

Venkova K., [45]	Conscious postop ileus ± morphine-treated rat model (male SD rats)	Ghrelin receptor agonist TZP-101 (Tranzyme Pharma Canada)	0.3–1 mg /Kg (1ml) IV given 1-2 min before meal	Assessment of IT by the transit of ^99m^Tc-labelled meal	TZP-101 accelerated IT dose-dependently in postop ileus rats ± morphine	

Zheng Q., [47]	Conscious diabetic mouse model (IP-alloxan-treated C57 mice)	GHRP-6 (Tocris, UK)	200 *μ*g/kg given IP prior to meal	Assessment of IT and CT by the transit of a phenol red-marked meal	GHRP-6 accelerated IT, but not CT; an effect blocked by pretreatment with atropine	GHRP-6 effects on intestinal motility involve the cholinergic pathway

Charoenthong-trakul S., [32]	Conscious opiate-induced bowel disorder mice model (male lean C57BL/6 mice)	Ghrelin receptor agonist EX-1314 (Elixir Pharma-ceuticals)	300 *μ*g/Kg given PO 5 min prior to meal	Assessment of IT by percentage of distance of charcoal travelled/total length of small intestine	EX-1314 reversed opiate-induced delayed IT	

Chen Y., [46]	LPS endotoxemia mouse model (male ICR mice)	Rat ghrelin (Global Peptide Services, UDA)	20 *μ*g/Kg IP given 15 min before meal	(i) Assessment of IT by the of distance charcoal travelled/total length of small intestine	(i) Ghrelin normalized endotoxemia-induced delayed IT	Ghrelin's effect on LPS-delayed IT transit is mediated via the down regulation of NO
				(ii) Assessment of plasma NO production by fluorometry	(ii) Ghrelin reduced plasma NO and iNOS expression in the submucosa and musculosa of the duodenum	
				(iii) Assessment of iNos expression by immunohisto-chemistry		

Fraser G., [68]	Conscious postop ileus rat model (male SD rats)	(i) Ghrelin receptor agonist TZP-101 (Tranzyme Pharma, Canada)	0.3–1 mg /Kg (t.i.d) IV given at 15 min, 2 and 4 h after surgery	Assessment of CT by monitoring the time of appearance and weight of fecal pellet output marked with trypan blue dye	TZP-101 accelerated CT dose-dependently at 12 and 24 h after surgery	

Venkova K., [69]	Conscious postop ileus rat model (male SD rats)	(i) Ghrelin receptor agonist and selective growth hormone secretagogue ipamorelin (Albany Molecular Research, Inc., NY)	(i) Ipamorelin 1 mg /Kg IV one dose or 0.1 repetitive doses	Assessment of CT by monitoring the time of appearance and weight of fecal pellet output marked with trypan blue dye	Ipamorelin and GHRP-6 accelerated CT 48 h after surgery	
		(ii) GHRP-6 (Sigma-Aldrich, MO)	(ii) GHRP-6 20 *μ*g/Kg IV bolus given after dosing of 4 doses/ day at 3 h intervals for 2 days after surgery			

## References

[1] Kojima M, Hosoda H, Date Y, Nakazato M, Matsuo H, Kangawa K (1999). Ghrelin is a growth-hormone-releasing acylated peptide from stomach. *Nature*.

[2] Tomasetto C, Wendling C, Rio MC, Poitras P (2001). Identification of cDNA encoding motilin related peptide/ghrelin precursor from dog fundus. *Peptides*.

[3] Peeters TL (2008). Old and new targets for prokinetic drugs: motilin and ghrelin receptors. *European Review for Medical and Pharmacological Sciences*.

[4] Poitras P, Tomasetto C (2009). The potential of ghrelin as a prokinetic. *Regulatory Peptides*.

[5] De Smet B, Mitselos A, Depoortere I (2009). Motilin and ghrelin as prokinetic drug targets. *Pharmacology and Therapeutics*.

[6] Sanger GJ (2008). Motilin, ghrelin and related neuropeptides as targets for the treatment of GI diseases. *Drug Discovery Today*.

[7] Taniguchi H, Ariga H, Zheng J, Ludwig K, Takahashi T (2008). Effects of ghrelin on interdigestive contractions of the rat gastrointestinal tract. *World Journal of Gastroenterology*.

[8] Camilleri M, Papathanasopoulos A, Odunsi ST (2009). Actions and therapeutic pathways of ghrelin for gastrointestinal disorders. *Nature Reviews Gastroenterology and Hepatology*.

[9] Venkova K, Greenwood-Van Meerveld B (2008). Application of ghrelin to gastrointestinal diseases. *Current Opinion in Investigational Drugs*.

[10] Tack J (2008). Prokinetics and fundic relaxants in upper functional GI disorders. *Current Opinion in Pharmacology*.

[11] Dass NB, Munonyara M, Bassil AK (2003). Growth hormone secretagogue receptors in rat and human gastrointestinal tract and the effects of ghrelin. *Neuroscience*.

[12] Bassil AK, Dass NB, Murray CD, Muir A, Sanger GJ (2005). Prokineticin-2, motilin, ghrelin and metoclopramide: prokinetic utility in mouse stomach and colon. *European Journal of Pharmacology*.

[13] Bassil AK, Dass NB, Sanger GJ (2006). The prokinetic-like activity of ghrelin in rat isolated stomach is mediated via cholinergic and tachykininergic motor neurones. *European Journal of Pharmacology*.

[14] Fukuda H, Mizuta Y, Isomoto H (2004). Ghrelin enhances gastric motility through direct stimulation of intrinsic neural pathways and capsaicin-sensitive afferent neurones in rats. *Scandinavian Journal of Gastroenterology*.

[15] Depoortere I, De Winter B, Thijs T, De Man J, Pelckmans P, Peeters T (2005). Comparison of the gastroprokinetic effects of ghrelin, GHRP-6 and motilin in rats in vivo and in vitro. *European Journal of Pharmacology*.

[16] Kitazawa T, De Smet B, Verbeke K, Depoortere I, Peeters TL (2005). Gastric motor effects of peptide and non-peptide ghrelin agonists in mice in vivo and in vitro. *Gut*.

[17] Levin F, Edholm T, Ehrstrom M (2005). Effect of peripherally administered ghrelin on gastric emptying and acid secretion in the rat. *Regulatory Peptides*.

[18] Kitazawa T, Kaiya H, Taneike T (2007). Contractile effects of ghrelin-related peptides on the chicken gastrointestinal tract in vitro. *Peptides*.

[19] Kitazawa T, Maeda Y, Kaiya H (2009). Molecular cloning of growth hormone secretagogue-receptor and effect of quail ghrelin on gastrointestinal motility in Japanese quail. *Regulatory Peptides*.

[20] Fujino K, Inui A, Asakawa A, Kihara N, Fujimura M, Fujimiya M (2003). Ghrelin induces fasted motor activity of the gastrointestinal tract in conscious fed rats. *Journal of Physiology*.

[21] Ariga H, Tsukamoto K, Chen C, Mantyh C, Pappas TN, Takahashi T (2007). Endogenous acyl ghrelin is involved in mediating spontaneous phase III-like contractions of the rat stomach. *Neurogastroenterology and Motility*.

[22] Tanaka R, Inui A, Asakawa A, Atsuchi K, Ataka K, Fujimiya M (2009). New method of manometric measurement of gastroduodenal motility in conscious mice: effects of ghrelin and Y2 depletion. *American Journal of Physiology*.

[23] Zheng J, Ariga H, Taniguchi H, Ludwig K, Takahashi T (2009). Ghrelin regulates gastric phase III-like contractions in freely moving conscious mice. *Neurogastroenterology and Motility*.

[24] Ohno T, Kamiyama Y, Aihara R (2006). Ghrelin does not stimulate gastrointestinal motility and gastric emptying: an experimental study of conscious dogs. *Neurogastroenterology and Motility*.

[25] Kobashi M, Yanagihara M, Fujita M, Mitoh Y, Matsuo R (2009). Fourth ventricular administration of ghrelin induces relaxation of the proximal stomach in the rat. *American Journal of Physiology*.

[26] Yin J, Chen J (2006). Inhibitory effects of gastric electrical stimulation on ghrelin-induced excitatory effects on gastric motility and food intake in dogs. *Scandinavian Journal of Gastroenterology*.

[27] Tumer C, Oflazoglu HD, Obay BD, Kelle M, Tasdemir E (2008). Effect of ghrelin on gastric myoelectric activity and gastric emptying in rats. *Regulatory Peptides*.

[28] Masuda Y, Tanaka T, Inomata N (2000). Ghrelin stimulates gastric acid secretion and motility in rats. *Biochemical and Biophysical Research Communications*.

[29] Trudel L, Tomasetto C, Rio MC (2002). Ghrelin/motilin-related peptide is a potent prokinetic to reverse gastric postoperative ileus in rat. *American Journal of Physiology*.

[30] Dornonville De La Cour C, Lindstrom E, Norlen P, Hakanson R (2004). Ghrelin stimulates gastric emptying but is without effect on acid secretion and gastric endocrine cells. *Regulatory Peptides*.

[31] Poitras P, Polvino WJ, Rocheleau B (2005). Gastrokinetic effect of ghrelin analog RC-1139 in the rat: effect on post-operative and on morphine induced ileus. *Peptides*.

[32] Charoenthongtrakul S, Giuliana D, Longo KA (2009). Enhanced gastrointestinal motility with orally active ghrelin receptor agonists. *Journal of Pharmacology and Experimental Therapeutics*.

[33] De Winter BY, De Man JG, Seerden TC (2004). Effect of ghrelin and growth hormone-releasing peptide 6 on septic ileus in mice. *Neurogastroenterology and Motility*.

[34] De Smet B, Depoortere I, Moechars D (2006). Energy homeostasis and gastric emptying in ghrelin knockout mice. *Journal of Pharmacology and Experimental Therapeutics*.

[35] Tack J, Depoortere I, Bisschops R (2006). Influence of ghrelin on interdigestive gastrointestinal motility in humans. *Gut*.

[36] Bisschops R (2008). Ligand and electrically induced acitivation patterns in myenteric neuronal networks. Confocal calcium imaging as a bridge between basic and human physiology. *Verhandelingen Koninklijke Academie voor Geneeskunde van België*.

[37] Cremonini F, Camilleri M, Vazquez Roque M (2006). Obesity does not increase effects of synthetic ghrelin on human gastric motor functions. *Gastroenterology*.

[38] Ang D, Nicolai H, Vos R (2009). Influence of ghrelin on the gastric accommodation reflex and on meal-induced satiety in man. *Neurogastroenterology and Motility*.

[39] Levin F, Edholm T, Schmidt PT (2006). Ghrelin stimulates gastric emptying and hunger in normal-weight humans. *Journal of Clinical Endocrinology and Metabolism*.

[40] Qiu W-C, Wang Z-G, Wang W-G, Yan J, Zheng Q (2008). Gastric motor effects of ghrelin and growth hormone releasing peptide 6 in diabetic mice with gastroparesis. *World Journal of Gastroenterology*.

[41] Qiu W-C, Wang Z-G, Wang W-G, Yan J, Zheng Q (2008). Therapeutic effects of ghrelin and growth hormone releasing peptide 6 on gastroparesis in streptozotocin-induced diabetic guinea pigs in vivo and in vitro. *Chinese Medical Journal*.

[42] Trudel L, Bouin M, Tomasetto C (2003). Two new peptides to improve post-operative gastric ileus in dog. *Peptides*.

[43] Liu Y-L, Malik NM, Sanger GJ, Andrews PLR (2006). Ghrelin alleviates cancer chemotherapy-associated dyspepsia in rodents. *Cancer Chemotherapy and Pharmacology*.

[44] Sallam HS, Oliveira HM, Gan HT, Herndon DN, Chen JDZ (2007). Ghrelin improves burn-induced delayed gastrointestinal transit in rats. *American Journal of Physiology*.

[45] Venkova K, Fraser G, Hoveyda HR, Greenwood-Van Meerveld B (2007). Prokinetic effects of a new ghrelin receptor agonist TZP-101 in a rat model of postoperative ileus. *Digestive Diseases and Sciences*.

[46] Chen YT, Tsai SH, Sheu SY, Tsai LH (2010). Ghrelin improves lipopolysaccharide-induced gastrointestinal motility disturbances: roles of nitric oxide and prostaglandin E2. *Shock*.

[47] Zheng Q, Qiu W-C, Yan J (2008). Prokinetic effects of a ghrelin receptor agonist GHRP-6 in diabetic mice. *World Journal of Gastroenterology*.

[48] Tack J, Depoortere I, Bisschops R, Verbeke K, Janssens J, Peeters T (2005). Influence of ghrelin on gastric emptying and meal-related symptoms in idiopathic gastroparesis. *Alimentary Pharmacology and Therapeutics*.

[49] Murray CDR, Martin NM, Patterson M (2005). Ghrelin enhances gastric emptying in diabetic gastroparesis: a double blind, placebo controlled, crossover study. *Gut*.

[50] Binn M, Albert C, Gougeon A (2006). Ghrelin gastrokinetic action in patients with neurogenic gastroparesis. *Peptides*.

[51] Ejskjaer N, Vestergaard ET, Hellstrom PM (2009). Ghrelin receptor agonist (TZP-101) accelerates gastric emptying in adults with diabetes and symptomatic gastroparesis. *Alimentary Pharmacology and Therapeutics*.

[52] Lasseter KC, Shaughnessy L, Cummings D (2008). Ghrelin agonist (TZP-101): safety, pharmacokinetics and pharmacodynamic evaluation in healthy volunteers: a phase I, first-in-human study. *Journal of Clinical Pharmacology*.

[53] Fraser GL, Hoveyda HR, Tannenbaum GS (2008). Pharmacological demarcation of the growth hormone, gut motility and feeding effects of ghrelin using a novel ghrelin receptor agonist. *Endocrinology*.

[54] Vestergaard ET, Gormsen LC, Jessen N (2008). Ghrelin infusion in humans induces acute insulin resistance and lipolysis independent of growth hormone signaling. *Diabetes*.

[55] Ankersen M, Kramer Nielsen K, Kruse Hansen T, Raun K, Sehested Hansen B (2000). Growth hormone secretagogues derived from NN703 with hydrazides as C-terminal. *European Journal of Medicinal Chemistry*.

[56] Edholm T, Levin F, Hellstrom PM, Schmidt PT (2004). Ghrelin stimulates motility in the small intestine of rats through intrinsic cholinergic neurons. *Regulatory Peptides*.

[57] Olsson C, Holbrook JD, Bompadre G (2008). Identification of genes for the ghrelin and motilin receptors and a novel related gene in fish, and stimulation of intestinal motility in zebrafish (Danio rerio) by ghrelin and motilin. *General and Comparative Endocrinology*.

[58] Wang Y, Dong L, Cheng Y, Zhao P (2007). Effects of ghrelin on feeding regulation and interdigestive migrating complex in rats. *Scandinavian Journal of Gastroenterology*.

[59] Date Y, Nakazato M, Murakami N, Kojima M, Kangawa K, Matsukura S (2001). Ghrelin acts in the central nervous system to stimulate gastric acid secretion. *Biochemical and Biophysical Research Communications*.

[60] Rauma J, Spangeus A, El-Salhy M (2006). Ghrelin cell density in the gastrointestinal tracts of animal models of human diabetes. *Histology and Histopathology*.

[61] Tebbe JJ, Mronga S, Tebbe CG, Ortmann E, Arnold R, Schafer MK-H (2005). Ghrelin-induced stimulation of colonic propulsion is dependent on hypothalamic neuropeptide Y1-and corticotrophin-releasing factor 1 receptor activation. *Journal of Neuroendocrinology*.

[62] Tebbe JJ, Tebbe CG, Mronga S, Ritter M, Schafer MKH (2005). Central neuropeptide Y receptors are involved in 3rd ventricular ghrelin induced alteration of colonic transit time in conscious fed rats. *BMC Gastroenterology*.

[63] Shimizu Y, Chang EC, Shafton AD (2006). Evidence that stimulation of ghrelin receptors in the spinal cord initiates propulsive activity in the colon of the rat. *Journal of Physiology*.

[64] Shafton AD, Sanger GJ, Witherington J (2009). Oral administration of a centrally acting ghrelin receptor agonist to conscious rats triggers defecation. *Neurogastroenterology and Motility*.

[65] De Smet B, Thijs T, Moechars D (2009). Endogenous and exogenous ghrelin enhance the colonic and gastric manifestations of dextran sodium sulphate-induced colitis in mice. *Neurogastroenterology and Motility*.

[66] Gonzalez-Rey E, Chorny A, Delgado M (2006). Therapeutic action of ghrelin in a mouse model of colitis. *Gastroenterology*.

[67] Konturek PC, Brzozowski T, Engel M (2009). Ghrelin ameliorates colonic inflammation. Role of nitric oxide and sensory nerves. *Journal of Physiology and Pharmacology*.

[68] Fraser GL, Venkova K, Hoveyda HR, Thomas H, Greenwood-Van Meerveld B (2009). Effect of the ghrelin receptor agonist TZP-101 on colonic transit in a rat model of postoperative ileus. *European Journal of Pharmacology*.

[69] Venkova K, Mann W, Nelson R, Greenwood-Van Meerveld B (2009). Efficacy of ipamorelin, a novel ghrelin mimetic, in a rodent model of postoperative ileus. *Journal of Pharmacology and Experimental Therapeutics*.

[70] http://www.tranzyme.com/.

